# The metabolic and circadian signatures of gestational diabetes in the postpartum period characterised using multiple wearable devices

**DOI:** 10.1007/s00125-024-06318-x

**Published:** 2024-11-12

**Authors:** Nicholas E. Phillips, Julie Mareschal, Andrew D. Biancolin, Flore Sinturel, Sylvie Umwali, Stéphanie Blanc, Alexandra Hemmer, Felix Naef, Marcel Salathé, Charna Dibner, Jardena J. Puder, Tinh-Hai Collet

**Affiliations:** 1 Service of Endocrinology, Diabetology, Nutrition and Therapeutic Education, Geneva University Hospitals, Geneva, Switzerland; 2 Laboratories of Neuroimmunology, Center for Research in Neuroscience and Service of Neurology, Department of Clinical Neurosciences, Lausanne University Hospital and University of Lausanne, Lausanne, Switzerland; 3 The Thoracic and Endocrine Surgery Division, Department of Surgery, Geneva University Hospitals, Geneva, Switzerland; 4 Department of Cell Physiology and Metabolism, Faculty of Medicine, University of Geneva, Geneva, Switzerland; 5 Gestational Diabetes Clinic, Service of Obstetrics, Department of Women-Mother-Child, Lausanne University Hospital and University of Lausanne, Lausanne, Switzerland; 6 Department of Rehabilitation and Geriatrics, Geneva University Hospitals, Geneva, Switzerland; 7 Diabetes Centre, Faculty of Medicine, University of Geneva, Geneva, Switzerland; 8 iGE3 Center, Geneva, Switzerland; 9 Department of Psychiatry, Addiction Medicine, Lausanne University Hospital and University of Lausanne, Lausanne, Switzerland; 10 Institute of Bioengineering, School of Life Sciences, EPFL (Ecole Polytechnique Fédérale de Lausanne), Lausanne, Switzerland; 11 Digital Epidemiology Lab, School of Life Sciences, School of Computer and Communication Sciences, EPFL (Ecole Polytechnique Fédérale de Lausanne), Lausanne, Switzerland

**Keywords:** Circadian rhythms, Continuous glucose monitoring, Gestational diabetes mellitus, Mean amplitude of glycaemic excursion, Postpartum period, Smartphone food diary app, Wearable devices

## Abstract

**Aims/hypothesis:**

Gestational diabetes mellitus (GDM) affects 14% of all pregnancies worldwide and is associated with cardiometabolic risk. We aimed to exploit high-resolution wearable device time-series data to create a fine-grained physiological characterisation of the postpartum GDM state in free-living conditions, including clinical variables, daily glucose dynamics, food and drink consumption, physical activity, sleep patterns and heart rate.

**Methods:**

In a prospective observational study, we employed continuous glucose monitors (CGMs), a smartphone food diary, triaxial accelerometers and heart rate and heart rate variability monitors over a 2 week period to compare women who had GDM in the previous pregnancy (GDM group) and women who had a pregnancy with normal glucose metabolism (non-GDM group) at 1–2 months after delivery (baseline) and 6 months later (follow-up). We integrated CGM data with ingestion events recorded with the smartphone app MyFoodRepo to quantify the rapidity of returning to preprandial glucose levels after meal consumption. We inferred the properties of the underlying 24 h rhythm in the baseline glucose. Aggregating the baseline and follow-up data in a linear mixed model, we quantified the relationships between glycaemic variables and wearable device-derived markers of circadian timing.

**Results:**

Compared with the non-GDM group (*n*=15), the GDM group (*n*=22, including five with prediabetes defined based on fasting plasma glucose [5.6–6.9 mmol/l (100–125 mg/dl)] and/or HbA_1c_ [39–47 mmol/mol (5.7–6.4%)]) had a higher BMI, HbA_1c_ and mean amplitude of glycaemic excursion at baseline (all *p*≤0.05). Integrating CGM data and ingestion events showed that the GDM group had a slower postprandial glucose decrease (*p*=0.01) despite having a lower proportion of carbohydrate intake, similar mean glucose levels and a reduced amplitude of the underlying glucose 24 h rhythm (*p*=0.005). Differences in CGM-derived variables persisted when the five women with prediabetes were removed from the comparison. Longitudinal analysis from baseline to follow-up showed a significant increase in fasting plasma glucose across both groups. The CGM-derived metrics showed no differences from baseline to follow-up. Late circadian timing (i.e. sleep midpoint, eating midpoint and peak time of heart rate) was correlated with higher fasting plasma glucose and reduced amplitudes of the underlying glucose 24 h rhythm (all *p*≤0.05).

**Conclusions/interpretation:**

We reveal GDM-related postpartum differences in glucose variability and 24 h rhythms, even among women clinically considered to be normoglycaemic. Our results provide a rationale for future interventions aimed at improving glucose variability and encouraging earlier daily behavioural patterns to mitigate the long-term cardiometabolic risk of GDM.

**Trial registration:**

ClinicalTrials.gov no. NCT04642534

**Graphical Abstract:**

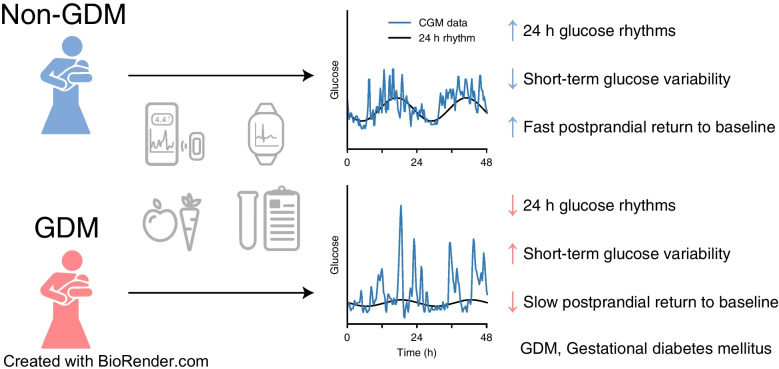

**Supplementary Information:**

The online version contains peer-reviewed but unedited supplementary material available at 10.1007/s00125-024-06318-x.


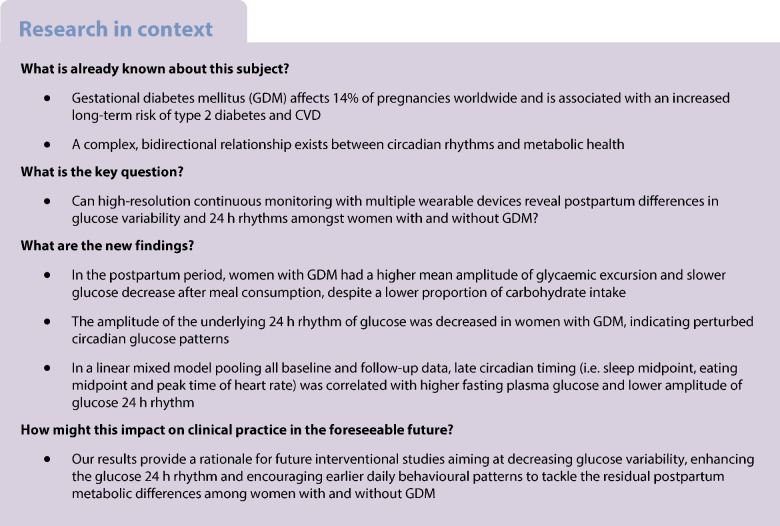


## Introduction

Gestational diabetes mellitus (GDM) is characterised by glucose intolerance in the second or third trimester of pregnancy that was not clearly overt diabetes prior to gestation [1]. While blood glucose levels typically normalise after delivery, women with GDM have up to tenfold higher risk of developing type 2 diabetes in the long term [2], as well as higher risk of developing the metabolic syndrome [3], CVD [4, 5] and kidney disease [6]. Considering that GDM affects approximately 10% of all pregnancies in Switzerland [7] and 14% worldwide [8], understanding the causes of elevated postpartum health risks and the risk-mitigation strategies represents a major health challenge.

Overweight, obesity, unhealthy diet and lack of exercise are important risk factors for the subsequent development of type 2 diabetes among women with GDM [9]. BMI and postpartum weight retention are major determinants of future type 2 diabetes risk [10]. Despite these established risk factors, the more precise physiological changes after GDM, even in the presence of a normoglycaemic clinical phenotype, and the role of the 24 h body clock remain unclear.

A new avenue to better identify the targets of lifestyle interventions is the use of wearable technologies that measure physiological signals in daily routine with high temporal resolution. Compared with glucose measurements at a single time point, continuous glucose monitoring can reveal the glucose dynamics in response to external perturbations such as carbohydrate-containing meals. This is particularly relevant for biomarkers of short-term glucose variability [11] that are hypothesised to contribute to diabetic complications through oxidative stress and endothelial function [12]. In parallel, the content and timing of each meal can be recorded by smartphone food diary applications. We recently published a computational method integrating ingestion events and data from continuous glucose monitors (CGMs) to model glucose levels as a combination of food-driven spikes and an underlying 24 h function [13]. The model parameters describe personalised features such as the postprandial glucose increases, the rapidity of returning to preprandial glucose levels after meal consumption, and the underlying glucose 24 h rhythm. While these parameters were previously quantified in a healthy population, it remains to be determined whether these fine-grained metrics can detect perturbed glucose dynamics in women with and without GDM in the postpartum period.

Circadian oscillators in the brain and in peripheral organs allow the body to anticipate daily environmental changes [14]. The circadian clock has a major impact on metabolism through energy balance, the endocrine system and direct regulation of metabolites [15–18], and circadian disruption is associated with type 2 diabetes in mouse models and humans [19, 20]. Wearable devices offer the capacity to quantify circadian physiological rhythmicity in free-living conditions. For example, sleep variables can be extracted from actigraphy data [21] and meal timing can be recorded using a smartphone application [22–24]. The timing of external behavioural cues such as sleep can influence glucose tolerance in individuals [25, 26], and later eating timing is associated with increased weight and cardiometabolic risk [27–29]. The in-depth investigation of the connection between circadian system changes and pathogenesis of metabolic diseases thus holds promise for refined diagnostics of metabolic diseases and developing personalised therapeutic approaches that consider the time dimension.

The objective of this study was to characterise the residual differences between GDM and non-GDM groups in the postpartum period across a range of emerging metabolic and circadian variables derived from multimodal wearable devices, including CGMs, a smartphone food diary app, physical activity and heart rate, in addition to standard clinical markers of glycaemic control. We aimed to identify differences between women with and without GDM at 1–2 months postpartum and 6 months later, and to characterise the relationships between circadian timing and glucose regulation in the postpartum period. We hypothesised that despite the clinically normoglycaemic phenotype in early postpartum, the fine-grained analysis with multimodal wearable devices could uncover differences in circadian timing and glucose regulation when comparing women with and without GDM.

## Methods

### Recruitment

In a prospective observational study at Lausanne University Hospital (CHUV) in Switzerland (ClinicalTrials.gov registration no. NCT04642534; Ethics Committee CER-VD no. 2019–01207), we recruited postpartum women who had GDM in the previous pregnancy (GDM group) and women who had a pregnancy with normal glucose metabolism (non-GDM group). GDM was diagnosed at 24–32 gestational weeks, according to the International Association of Diabetes and Pregnancy Study Groups consensus criteria [30]. Information on the study was provided during their antenatal appointment at the Maternity clinic or at the end of their pregnancy. Each participant gave written consent in accordance with the Declaration of Helsinki. The recruitment was conducted from February 2020 to June 2022 and was paused intermittently during the COVID-19 pandemic as pregnant and postpartum women were considered at risk and access to research facilities was not permitted.

**Fig. 1:**
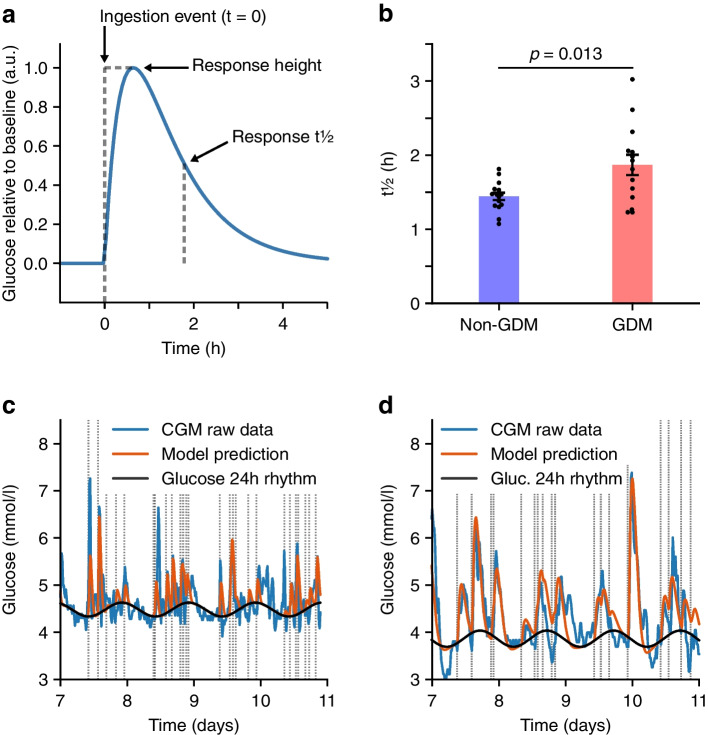
Dynamical modelling of glucose levels reveals differences in glucose response to ingestion events. (**a**) Schematic illustrating how a shorter glucose t½ leads to a faster return to baseline levels after ingestion. (**b**) Comparison of glucose t½ between non-GDM (blue) and GDM (red) groups. Each data point represents a unique participant. (**c**, **d**) Examples of participants with a short (**c**, participant ID 03, non-GDM group, fast dynamics) and long (**d**, participant ID 29, GDM group, slow dynamics) glucose t½. Blue, CGM raw data; orange, model prediction incorporating ingestion events and the underlying 24 h glucose rhythm; black, the underlying 24 h glucose rhythm; dashed lines, meal time-stamps. a.u., arbitrary unit

We included mothers at 1–2 months postpartum, who were aged 18–40 years, were breastfeeding at inclusion and were confident users of a smartphone compatible with the MyFoodRepo app (iOS, Android) [23, 31] able to take regular pictures of food/drinks. We excluded women with pre-existing diabetes (i.e. diagnosed prior to the pregnancy), or with a major illness/fever, who were enrolled in another study, planned for shift work after maternity leave, or unable to give informed consent or follow the study procedures. At baseline, some women were diagnosed with prediabetes but were not excluded from the study nor treated pharmacologically. The eligibility criteria and reasons for exclusion are detailed in the study flowchart (electronic supplementary material [ESM] Fig. 1).

Measurements were conducted at two main time points: at baseline in the early postpartum period (i.e. between 1 and 2 months after delivery [visits 1 and 2, including continuous measurements during the 2 week interval]); and at follow-up 6 months later (i.e. at 7–8 months after delivery [visits 4 and 5 and the 2 week interval]). Between both time points, we made a phone call (visit 3) to gather information from additional questionnaires and obtain self-reported weight.

### Clinical measurements

We recorded data on demographics, lifestyle (smoking status and physical activity using the short form of International Physical Activity Questionnaire [32]), chronotype (Morningness–Eveningness Questionnaire [33]), sleep duration and times (adapted from the Pittsburgh Sleep Quality Index [34]), anthropometrics, BP, fasting plasma glucose, HbA_1c_ and lipid profile. Prediabetes was defined as a fasting plasma glucose 5.6–6.9 mmol/l (100–125 mg/dl) and/or HbA_1c_ 39–47 mmol/mol (5.7–6.4%) at baseline [1]. See ESM Methods (Clinical measurements) for further details.

### Wearable device data

Clinical measurements and questionnaire data were complemented with data from wearable devices (i.e. the smartphone food diary app MyFoodRepo to record time-stamped food and drink consumption), CGM and a triaxial accelerometer monitoring physical activity, heart rate and sleep. For each participant, we collected data using the following devices over 2 weeks at each time point.

The smartphone app MyFoodRepo recorded the time-stamps along with pictures, barcodes of packaged items, or free text descriptions of all consumed food items and drinks [23, 31]. The macronutrient composition of each consumed item was automatically extracted from a constantly updated nutritional composition table, then verified by trained dietitians. The eating duration was defined as the time interval between the 2.5th and the 97.5th percentiles of all time-stamped ingestion events over 2 weeks, and the eating midpoint was defined as the 50th percentile. Continuous glucose monitoring data was recorded with an Abbott FreeStyle Libre Pro device for 2 weeks; this device reports interstitial glucose every 15 mins. CGM metrics related to glycaemic control were extracted from the CGM data [35], where we focused on the mean glucose level, CV (SD/mean) and the mean amplitude of glycaemic excursions (MAGE) [36]. Actigraphy was assessed using the triaxial accelerometer GENEActiv (Activinsights, UK). Data on daily activity and sleep–wake cycles were analysed with the GGIR package in R (v4.0.0) [21], and we extracted the sleep midpoint and sleep duration. The CamNTech ActiHeart device (version 4) assessed physical activity (activity counts [proprietary algorithm]), heart rate (beats/min) and heart rate variability (using root mean square of successive differences [1/RMSSD] between normal heartbeats during 24–72 h at each time point [37]). See ESM Methods (Wearable device data) for details.

### Statistical analyses

Continuous variables are reported as mean ± SD and compared with unpaired two sample *t* test, using the Welch separate variances *t* test when sample sizes are different [38] using the ‘pingouin’ package (v0.5.3) within Python, available from conda-forge at https://anaconda.org/conda-forge/pingouin. Non-normally distributed continuous variables (e.g. serum lipids and MAGE) are reported as median (IQR) and compared with the Mann–Whitney *U* test (i.e. Wilcoxon rank-sum test). Categorical variables are presented as number of participants (% in group) and compared using the Fischer exact test. We performed a sensitivity analysis according to prediabetes status.

Associations between clinical, questionnaire and wearable device variables with glycaemic variables were quantified by combining the baseline and follow-up data within a linear mixed regression model accounting for repeated data in individual participants. The aim of the random effect was to account for potential correlations within the same participants, and the model was fitted using the ‘MixedLM’ class from the ‘statsmodels’ library (v0.13.0), available from conda-forge at https://anaconda.org/conda-forge/statsmodels. We performed a complete-case analysis using all available data and hence no imputation methods were used for missing data.

#### Modelling of ingestion events and glucose data

**Fig. 2:**
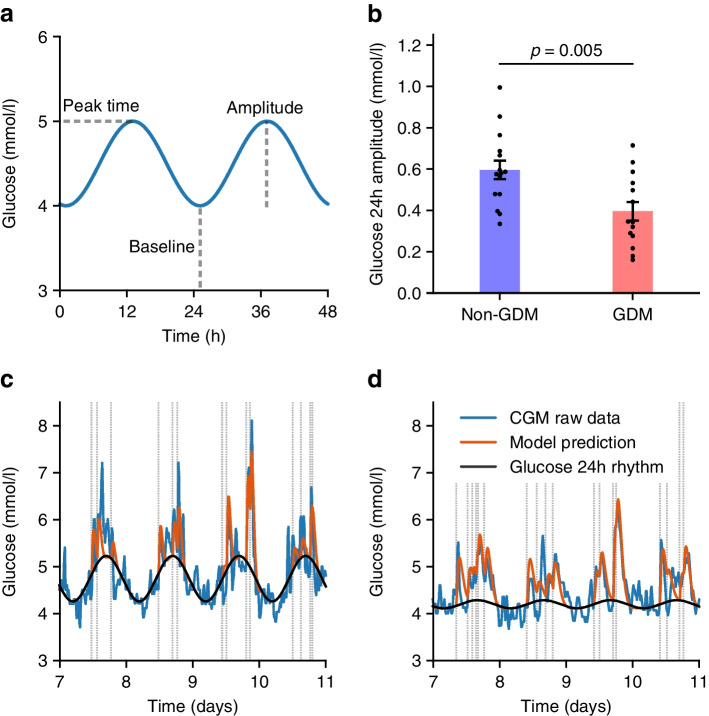
Dynamical modelling of glucose levels reveals differences in the underlying 24 h glucose rhythm. (**a**) Schematic illustrating the parameters of the underlying 24 h glucose rhythm. (**b**) Comparison of 24 h glucose amplitude between non-GDM (blue) and GDM (red) groups. Each data point represents a unique participant. (**c**, **d**) Examples of participants with a high amplitude (**c**, participant ID 05, non-GDM group) and low amplitude (**d**, participant ID 17, GDM group) in 24 h glucose rhythm. Blue, CGM raw data; orange, model prediction incorporating ingestion events and the underlying 24 h glucose rhythm; black, the underlying 24 h glucose rhythm; dashed lines, meal time-stamps

We combined the ingestion events with CGM data based on a recently published computational framework to extract individual parameters related to glucose responses to ingestion events (Fig. 1a) [13]. The MyFoodRepo app provided a list of time-stamps of each consumed food and drink (depicted at time t=0 in Fig. 1a). The increases in glucose caused by meals (termed ‘response heights’, Fig. 1a) were left as free parameters which were fit to the data during the model inference. After glucose increase, the model also learnt a characteristic ‘response t½’ (Fig. 1a) describing how long the glucose takes to return to baseline. Even when postprandial glucose spikes caused by meals were accounted for in the model, the meal model alone left systematic 24 h trends in the model residual (see ESM Results), thus an underlying 24 h cosinor function was also included. The 24 h cosinor function is described by ‘baseline’, ‘peak-to-trough amplitude’ and ‘peak time’ parameters (schematic in Fig. 2a), which were also learnt for each participant. See ESM Methods (Statistical analyses) for further details.

#### Cosinor analysis of Actiheart and GENEActiv data

As the time-length of data was shorter for Actiheart than for the other wearable devices (between 2 and 4 days), we used a simplified approach based on cosinor regression with a fixed period of 24 h. The model for the cosinor function is as follows:

$$y(t) ={A}_{0}+{A}_{1}(1+{\text{cos}}(w(t-\phi )))/2$$

where $y(t)$ represents the Actiheart signal (either activity counts, heart rate or heart rate variability), *A*_0_ is the baseline, *A*_1_ is the amplitude, *w* is the frequency (fixed to give a period of 24 h) and *Φ* is the peak time. We fit this function to the data by minimising the least squared error using the ‘curve_fit’ function within SciPy (v1.7.3), available from conda-forge at https://anaconda.org/conda-forge/scipy. We also applied this approach to the GENEActiv activity data.

No sample size estimation was performed prior to the study due to its pilot nature in this specific population of postpartum women. We initially aimed at 15 participants in each group and continued recruitment to account for withdrawals, loss to follow-up and missing data. A *p* value below 0.05 was considered significant and no adjustment for multiple testing was performed in this exploratory study.

## Results

Among 199 individuals screened for participation, 22 were included in the GDM group and 15 in the non-GDM group (for study flowchart, see ESM Fig. 1). The age, education level, smoking status and mode of breastfeeding were not statistically different between the two groups (Table 1).

**Table 1:** Baseline clinical characteristics of the GDM and non-GDM groups

Characteristic	GDM group (*n*=22)	Non-GDM group (*n*=15)	*p* value
Demographics			
Age, years	32.7±4.1	30.3±5.5	0.19
Education			0.37
Apprenticeship	7 (31.8)	4 (26.7)	
College/High school	4 (18.2)	1 (6.7)	
Professional school	3 (13.6)	6 (40.0)	
University/University of applied sciences	8 (36.4)	4 (26.7)	
Smoking status			0.26
Current	4 (18.2)	0 (0.0)	
Past	5 (22.7)	5 (33.3)	
Never	13 (59.1)	10 (66.7)	
Parity			0.68
1	13 (59.1)	10 (66.7)	
2	8 (36.4)	5 (33.3)	
≥3	1 (4.5)	0 (0.0)	
Breastfeeding			0.51
Exclusive breastfeeding	12 (54.5)	10 (66.7)	
Mixed (breastfeeding + bottle)	10 (45.5)	5 (33.3)	
Clinical variables			
BMI, kg/m^2^	28.7±5.6	25.0±4.2	0.03
Waist circumference, cm	93.5±14.6	85.7±9.0	0.06
WHR	0.86±0.06	0.83±0.05	0.20
Systolic BP, mmHg	111.8±15.1	116.5±9.1	0.26
Diastolic BP, mmHg	72.5±9.1	76.7±7.0	0.13
Fasting plasma glucose, mmol/l	4.9±0.5	4.7±0.3	0.07
HbA_1c_, mmol/mol (IFCC)	34.9±4.3	32.0±3.0	0.03
HbA_1c_, % (DCCT)	5.3±0.4	5.1±0.3	0.03
Prediabetes^a^	5 (22.7)	0 (0.0)	0.07
Total cholesterol, mmol/l	5.4 (4.7–5.9)	5.8 (4.8–6.2)	0.39
HDL-cholesterol^b^, mmol/l	1.5 (1.3–1.8)	1.8 (1.4–1.8)	0.56
LDL-cholesterol^b^, mmol/l	3.2 (2.6–3.7)	3.6 (2.9–4.1)	0.30
Triacylglycerols, mmol/l	1.1 (0.8–1.4)	0.9 (0.8–1.1)	0.28

Data are presented as means ± SD, or medians (IQR) if not normally distributed, or *n* (% in group) for categorical variables

^a^Prediabetes was defined as a fasting plasma glucose 5.6–6.9 mmol/l (100–125 mg/dl) and/or HbA_1c_ 39–47 mmol/mol (5.7–6.4%)

^b^In the non-GDM group, two participants had no measured HDL-cholesterol and thus no calculated LDL-cholesterol levels

### Clinical measurements show increased metabolic dysfunction in the GDM group at 1–2 months postpartum

At baseline (1–2 months postpartum), the mean BMI was different between the GDM and non-GDM groups (mean difference +3.66 kg/m^2^ [95% CI 0.29, 7.03], *p*=0.03; Table 1); HbA_1c_ also differed between groups (mean difference +2.85 mmol/mol [95% CI 0.35, 5.35 mmol/mol] or +0.26% [95% CI 0.03, 0.49%], *p*=0.03; Table 1 and ESM Fig. 2a). The difference in BMI and HbA_1c_ was no longer significant in a sensitivity analysis without the five women with GDM and prediabetes at the first study visit. The other clinical measurements were not different between groups (Table 1).

### Increased energy intake from fat and reduced energy intake from carbohydrates in the GDM group

Energy intake from carbohydrates was higher in the non-GDM group (mean ± SD 44.9±5.6%) than in the GDM group (mean ± SD 39.4±6.0%, *p*=0.02), while the energy intake from fat was lower in the non-GDM group (mean ± SD 39.0±4.2%) than in the GDM group (mean ± SD 43.5±5.9%, *p*=0.03; Table 2 and ESM Fig. 2b).

**Table 2:** Comparison of GDM and non-GDM groups across food and drink consumption measured with the smartphone app, CGM metrics and parameters extracted with the glucose model

Characteristic	GDM group	Non-GDM group	*p* value
Food and drink consumption	(*n *=15)	(*n *=15)	
Carbohydrates, % of energy intake	39.4±6.0	44.9±5.6	0.02
Protein, % of energy intake	17.1±3.4	16.0±3.2	0.40
Fat, % of energy intake	43.5±5.9	39.0±4.2	0.03
Energy intake, kJ/day (kcal/day)	6538±2417 (1562.5±577.8)	7352±1404 (1757.1±335.5)	0.29
Eating duration, h	13.7±1.4	13.6±1.6	0.74
Eating midpoint, h	14.9±1.3	14.6±1.8	0.67
CGM metrics	*n *=17	*n *=15	
Mean glucose, mmol/l	4.5±0.4	4.4±0.5	0.36
CV, %	16.8±3.9	15.0±2.6	0.14
MAGE, mmol/l	1.46 (1.32–1.69)	1.26 (1.08–1.38)	0.04
Glucose model parameters	*n *=14	*n *=15	
Baseline, mmol/l	4.0±0.4	3.8±0.4	0.24
24 h amplitude, mmol/l	0.4±0.2	0.6±0.2	0.005
Response t½, h	1.9±0.5	1.4±0.2	0.01
Mean response height, mmol/l	0.7±0.2	0.5±0.2	0.07

Data are presented as means ± SD, or as medians (IQR) if not normally distributed

*p* values were calculated using Welch separate variances *t* test for continuous variables, except for MAGE, which was compared with the Mann–Whitney *U* test

The chrononutrition-related metrics derived from the MyFoodRepo app showed that the mean eating window and eating midpoint were similar between groups (Table 2). However, these values showed associations with glycaemic variables in a continuous analysis pooling data from both groups and time points (see below).

### Higher glucose variability in the GDM group, even in the absence of prediabetes

While the mean glucose and CV calculated from the CGM data were not different between groups at baseline (both *p*≥0.14; Table 2 and ESM Fig. 2c), MAGE was higher in the GDM group (median 1.46 [IQR 1.32–1.69] mmol/l) than in the non-GDM group (median 1.26 [IQR 1.08–1.38] mmol/l,* p*=0.04). Furthermore, when women with prediabetes at baseline were excluded in a sensitivity analysis (5/22 in the GDM group), the difference in MAGE persisted (*p*=0.02).

Comparing the mean 24 h glucose profiles between groups did not reveal a clear signal due to high interindividual heterogeneity (ESM Fig. 3). This motivated the use of a computational model combining ingestion events and CGM data to better characterise the glucose dynamics for each participant.

### Glucose decrease after meal consumption is slower in the GDM group

Figure 1a shows a schematic of the glucose dynamics model, which estimates the height of the glucose spikes (termed ‘response heights’) after food or drink consumption, as well as the time required for glucose to return to baseline after a spike (termed ‘response t½’). Among these glucose model parameters (Table 2), the response t½ was ~30 min longer (i.e. slower postprandial decrease of glucose) in the GDM group than in the non-GDM group at baseline (*p*=0.01, Fig. 1b); this was in the absence of higher carbohydrate intake in women with GDM (Table 2). As an example, the participant with the shortest response t½ (participant ID 03, who was in the non-GDM group) showed rapid responses to meals, with glucose returning quickly to baseline levels after meals (Fig. 1c). In contrast, the participant with the longest t½ (ID 29, who was in the GDM group) showed comparatively much slower glucose responses, where glucose excursions visibly took much longer to return to baseline levels after ingestion events (Fig. 1d).

### Lower amplitude of the underlying glucose 24 h rhythm in the GDM group

The glucose dynamics model also provides the underlying glucose 24 h rhythm (schematic shown in Fig. 2a), with the amplitude being higher in the non-GDM group than in the GDM group at baseline (i.e. with more pronounced oscillations around the 24 h clock [*p*=0.005, Table 2, Fig. 2b]). The participant with the highest amplitude showed a regular underlying 24 h rhythm, with ingestion events causing further glucose spikes on top of the oscillating trendline (ID 05, who was in the non-GDM group, Fig. 2c), while this 24 h rhythm was barely detectable in the participant with the lowest amplitude (ID 17, who was in the GDM group, Fig. 2d). The amplitude of the underlying glucose 24 h rhythm did not affect overall glucose variability. (see ESM Results and ESM Fig. 4 for further details).

### Fasting glucose and cholesterol evolve during the postpartum period

We assessed the evolution of clinical and glycaemic variables from baseline (1–2 months postpartum; median 42 [range 26–65] days since delivery) to follow-up (6 months later; median 221 [range 200–285] days since delivery). At follow-up, there was no difference in breastfeeding mode between groups (*p*=0.45). The comparison across both groups revealed a decrease in waist circumference, total cholesterol and LDL-cholesterol (all *p*≤0.001) and an increase in fasting plasma glucose (*p*=0.02) over time (ESM Fig. 5a), but no between-group differences in these changes (ESM Fig. 5b) (see ESM Results for further details).

### Glycaemic variables are correlated with sleep behaviour and ‘eveningness’ across both groups

**Fig. 3:**
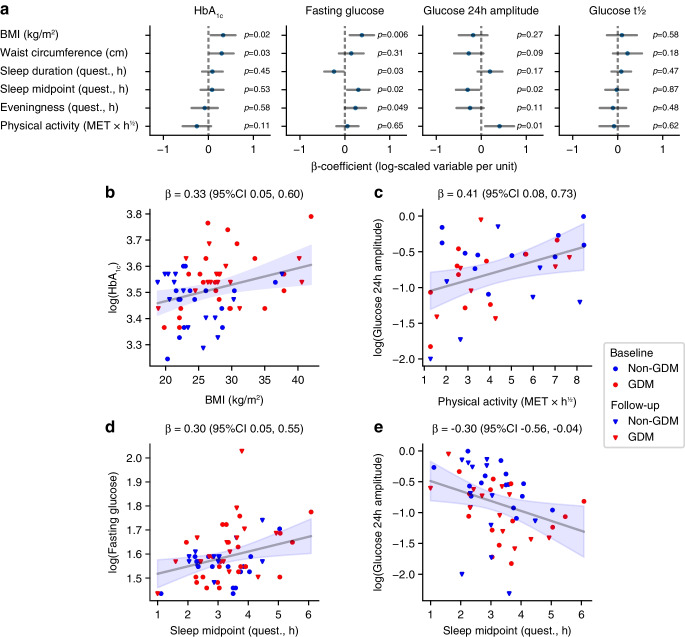
In a linear mixed model pooling all baseline and follow-up data, glycaemic variables are associated with clinical measurements and lifestyle factors as measured with questionnaires. (**a**) Points show the inferred regression coefficient, horizontal lines the 95% CI; *p* values are also shown. The dependent variables were log-transformed (natural log). (**b**–**e**) Examples of regressions for specific variables. β denotes the inferred regression coefficient (with 95% CI), which is also shown graphically (grey line and shaded blue, respectively). Blue symbols, non-GDM group; red symbols, GDM group; circles, baseline; triangles, follow-up. MET, metabolic equivalent of task; quest, questionnaire

Pooling baseline and follow-up data from both groups to increase power in a linear mixed model confirmed the known association between BMI and both HbA_1c_ and fasting plasma glucose (Fig. 3a, b). We also found that a high amplitude of glucose 24 h rhythm was associated with more physical activity (as measured with the International Physical Activity Questionnaire, *p*=0.01, Fig. 3c), high HDL-cholesterol and low triacylglycerols (both *p*≤0.004, ESM Fig. 6).

We then explored the relationships between glycaemic variables and sleep behaviour and chronotype as assessed with questionnaires. Late sleep midpoint was associated with higher fasting plasma glucose and weaker amplitudes in the underlying 24 h glucose rhythm (both *p*=0.02, Fig. 3d, e). The eveningness score and a short sleep duration were associated with higher fasting plasma glucose (*p*=0.049 and 0.03, respectively, Fig. 3a).

### Wearable device-derived markers of late circadian timing are associated with higher fasting plasma glucose and lower amplitude of glucose 24 h rhythm

**Fig. 4:**
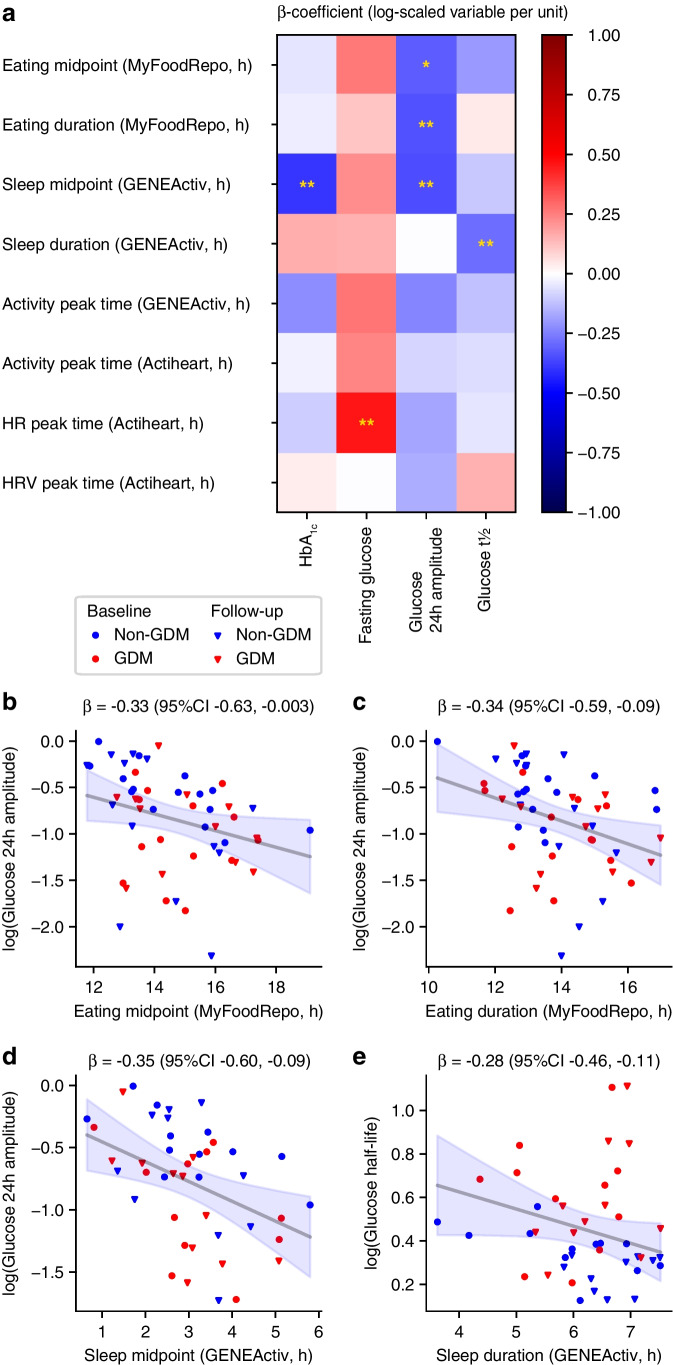
The relationship between wearable device-derived timing metrics and glycaemic variables. (**a**) The β regression coefficients between glycaemic and timing variables are shown as a heat map. The dependent variables were log-transformed (natural log). *p<0.05, **p<0.01. (**b**–**e**) Examples of regressions for specific variables. β denotes the inferred regression coefficient (with 95% CI), which is also shown graphically (grey line and shaded blue, respectively). Blue symbols, non-GDM group; red symbols, GDM group; circles, baseline, triangles, follow-up. HR, heart rate; HRV, heart rate variability

Finally, we again pooled baseline and follow-up data within a linear mixed model framework to quantify the relationships between circadian timing metrics derived from the MyFoodRepo, GENEActiv and Actiheart wearables and glycaemic variables (Fig. 4a).

The eating midpoint (i.e. the daily midpoint of all food and drink consumption) and eating duration were similar between non-GDM and GDM groups at baseline (Table 2). However, regression analysis across both time points revealed that an early eating midpoint and shorter eating duration were correlated with a higher amplitude glucose 24 h rhythm (*p*=0.048 and *p*=0.008, respectively, Fig. 4b, c).

A similar analysis of wearable device-derived sleep metrics showed that a late sleep midpoint was associated with a lower amplitude glucose 24 h rhythm (Fig. 4d), and shorter sleep was associated with a longer glucose response t½ (Fig. 4e). Among the heart activity timing metrics, the strongest association was between a later peak time of the heart rate and higher fasting plasma glucose (Fig. 4a).

## Discussion

Combining clinical, questionnaire and wearable device data to better characterise differences in glucose regulation, our study shows residual differences between GDM and non-GDM groups in the postpartum period, even when investigating only women with GDM and a normoglycaemic phenotype according to standard criteria. Women with GDM had a higher MAGE, a slower glucose decrease after meal consumption despite a tendency for a lower carbohydrate intake, and a reduced amplitude of the underlying glucose 24 h rhythm. The continuous monitoring with multiple wearable devices highlighted that late circadian timing was correlated with poorer glycaemic control. A better and earlier detection of the metabolic risk of GDM could improve targeting of lifestyle measures in this population at risk, including possible interventions targeting the circadian rhythms.

In contrast to HbA_1c_, the increased MAGE between GDM and non-GDM women at 1–2 months postpartum remained significant when the five individuals with prediabetes were excluded from analysis. MAGE quantifies short-term glucose variability and is associated with impaired early-phase insulin secretion in GDM [39]. It is also associated with oxidative stress that could contribute to the complications of type 2 diabetes [12] and atherosclerosis [40]. An elevated MAGE has been observed among Asian women after GDM [41], and was also associated with decreased beta cell function. However, Asian populations might show specificities regarding beta cell function [42] compared with our study of mostly European descent. Thus, MAGE could be an early marker of reduced beta cell function, even in the absence of differences in standard glycaemic variables.

The integration of the ingestion events with CGM data showed a 30 min longer mean glucose decrease after meal consumption in the GDM group. One explanation could be that higher GDM-related insulin resistance persists in the postpartum period [43]. Reduced rates of insulin secretion could also contribute to this slower decrease in postprandial glucose [44, 45]. Both these hypotheses, given the absence of differences in fibre intake or in physical activity, could explain the increased risk of progression to type 2 diabetes. We propose that using finer tools with high temporal resolution and modelling of postprandial glucose response could better characterise the underlying metabolic state of GDM in women who would not be considered prediabetic by standard criteria [1, 46].

There is an intricate and bidirectional relationship between circadian clocks and metabolic health [47, 48], and circadian clocks in metabolic organs are essential regulators of glucose and lipid homeostasis [18, 49]. Our study presents two key insights into circadian rhythms in the postpartum period. First, the amplitude of the underlying glucose 24 h oscillations was less pronounced in the GDM group, which might indicate an early perturbation of circadian rhythms in this population. This differential rhythmic activity could be caused by differences in glucose processing kinetics and linked to the central circadian clock in the suprachiasmatic nucleus [50] or the peripheral circadian clocks [51]. Second, we found multiple associations between the glucose 24 h rhythm and behavioural patterns. We observed that a high amplitude of the glucose 24 h rhythm was associated with a shorter eating duration and higher self-reported physical activity. The combination of wearable device data and questionnaire data showed that a lower amplitude of glucose 24 h rhythm was also associated with later sleep midpoint, later eating midpoint and higher eveningness score and that higher fasting plasma glucose correlated with a later peak time of heart rate. The preference for morning vs evening is often termed ‘chronotype,’ and a late chronotype has been linked with increased risk of diabetes and the metabolic syndrome [52, 53], and a poorer glycaemic control in those with type 2 diabetes [54]. As such, the amplitude of the underlying glucose 24 h rhythm could represent a novel biomarker delineating GDM and non-GDM groups, and future studies should better characterise its mechanistic underpinning and relationships with lifestyle variables.

The in-depth profiling of the metabolic and circadian signatures of women in the postpartum period with wearable devices enabled the characterisation of glucose variability and 24 h patterns.

Nonetheless, the study has a few limitations. First, the study may be underpowered to discriminate between the two groups due to the small sample size. For example, in accordance with the elevated MAGE and glucose response t½, the postprandial meal spikes and glucose CV were slightly higher in the post-GDM group, but these differences were not statistically significant. The study explored many wearable device-derived metrics simultaneously but, now that the most promising biomarkers have been identified, we aim to focus on a more restricted set of variables in follow-up studies. Second, we recruited women with GDM and normal glycaemic control at a single tertiary centre. Because most were of mostly European descent, not all results may apply to other populations and settings, impacting the external validity of this study. Third, while the energy intake per day was not significantly different between groups, the extent of missing food and drink data is difficult to assess. Finally, while the wearable device data revealed differences between the GDM and non-GDM groups in the postpartum period, we were unable to determine the precise moment at which these differences arose. Furthermore, the extent to which these differences are driven by lifestyle factors such as diet or by differences in BMI also remains to be fully elucidated.

Our study emphasises postpartum differences between GDM and non-GDM groups, encompassing elevated short-term glucose variability (MAGE), slower postprandial glucose response and a weaker amplitude of glucose 24 h rhythm. Combining data across all time points and groups, the longitudinal regression analysis highlighted the relationship between late chronotype and poorer glycaemic health in the postpartum period. The major clinical implication is the uncovering of biomarkers of metabolic health that may not be detected by current standard measurements in the postpartum period. Our results suggest that future interventions based on earlier meal timing, a shorter eating window and an earlier sleep midpoint may be useful for improving glycaemic control in the postpartum period and thus mitigate the long-term cardiometabolic risk of GDM. We propose wearable technologies to assess the adherence and individual response to interventions that would complement the standard clinical measurements and questionnaires in trials evaluating personalised lifestyle measures in people at increased metabolic risk.

## Supplementary Information

Below is the link to the electronic supplementary material.

ESM (PDF 1.59 MB)

## Data Availability

The data are available upon reasonable request to the corresponding authors.

## Abbreviations

CGM: Continuous glucose monitors
GDM: Gestational diabetes mellitus
MAGE: Mean amplitude of glycaemic excursions
