# Supplementary material for: The metabolic and circadian signatures of gestational diabetes in the postpartum period characterised using multiple wearable devices
Source: Diabetologia. 2024 Nov 12;68(2):419–32. doi: 10.1007/s00125-024-06318-x (PMC11732869; doi:10.1007/s00125-024-06318-x)
Supplement: Supplementary file 1 — ESM (PDF 1.59 MB) [file 125_2024_6318_MOESM1_ESM.pdf]

Electronic Supplementary Material

Phillips *et al*. The metabolic and circadian signatures of gestational diabetes in the postpartum period characterised using multiple wearable devices

ESM Methods

Clinical measurements

We recorded demographics (e.g. age, socio-economic status), information on the previous pregnancies and deliveries (e.g. gestational age, breastfeeding and use of infant formula), and lifestyle (smoking status, physical activity using the short form of International Physical Activity Questionnaire [1]).

We assessed the self-reported sleep duration, sleeping times, sleep quality (Pittsburgh Sleep Quality Index [2]), and chronotype (Morningness-Eveningness Questionnaire [MEQ] [3], ranging from definitely morning type [70–86 points], moderately morning type [59–69 points], neutral [42–58 points], moderately evening type [31–41 points], to definitely evening type [16–30 points]). Eveningness was calculated by taking the negative value of the MEQ score for ease of presentation in the comparisons with sleeping times.

Weight was measured in light clothing and the body mass index (BMI) was calculated as the weight in kilograms divided by the height in meters squared. Blood pressure was measured three times with a calibrated monitor (Omron Intellisense BP monitor, Omron Healthcare) and an appropriately sized arm cuff, after 5 min of rest in the sitting position, and the last two values were averaged. Waist and hip circumferences, and waist-to-hip ratio (WHR) were assessed according to the WHO standard procedure [4].

Fasting plasma glucose, glycated hemoglobin (HbA_{1c}), lipid profile (total, HDL and LDL cholesterol, triacylglycerols) were measured with the usual assays at the clinical lab of Lausanne University Hospital. Prediabetes was defined as a fasting plasma glucose 5.6–6.9 mmol/L (100–125 mg/dL) and/or HbA1c 39–47 mmol/mol (5.7–6.4%) [5] after inclusion. Thus the stratified analysis by prediabetes status was added *post hoc* in the study.

Wearable device data

Clinical measurements and questionnaire data were complemented with data from wearable devices, such as the MyFoodRepo smartphone application (app) to record timestamped food/drink consumption, Continuous Glucose Monitoring (CGM, Abbott FreeStyle Libre Pro), as well as physical activity, heart and sleep monitoring (GENEActiv + Actiheart). For each participant, we collected data using the following devices over 2 weeks at each time point.

<u>The smartphone app MyFoodRepo</u> recorded the timestamps and pictures or barcodes of all consumed food items and drinks [6, 7]. Each picture was reviewed by a trained dietician assisted by machine learning to estimate the quantity of each food component. The caloric and macronutrient composition of each consumed item was automatically extracted from a constantly updated nutritional composition table. This could be completed with a remote chat function with the participant when additional information was needed. This novel method to measure human eating behavior in free-living settings is less intrusive and reduces the burden of writing down each ingestion event for two weeks, given the everyday use of smartphones in all populations [6–9]. Optionally, participants could type text-only entries, e.g. if the smartphone ran out of battery, or if it was not socially acceptable to take pictures in the current context. Macronutrient consumption is reported in terms of percentages of total energy intake instead of macronutrients in grams to minimize the effect of missing ingestion entries. The eating duration was defined as the time interval between the 2.5th and the 97.5th percentiles of all time stamped ingestion events over 2 weeks, and the eating midpoint

was defined as the 50th percentile. To account for the social consumption habits and the nadir of food entries, we calculated the percentiles using a start time of 04:00 as defined previously [8, 10].

CGM was recorded with an Abbott FreeStyle Libre Pro device for two weeks, which reports interstitial glucose every 15 minutes. The package rGV in R was used to extract metrics related to glycemic control from the CGM data [11], where we focused on the mean glucose level, coefficient of variation (CV, standard deviation / mean), and the mean amplitude of glycemic excursions (MAGE). MAGE is calculated using the mean differences from peaks to nadirs in the CGM time-series data [12], and it is a measure of short-term within-day glucose variability. During the first 24-36 hour period of recording, the CGM data can occasionally spend the majority of the time at the device minimal value of 2.2 mmol/L due to the transient effects of micro-trauma, local pressure leading to lower blood flow and device calibration, and in this case we removed the initial data until the device exited this floor state. As a preliminary step of glucose dynamics modeling (see below), abnormally low glucose values (that are typical of the early recording hours) are also corrected for by removing long timescale trends within the time series.

Actigraphy was assessed using a triaxial accelerometer (GENEActiv, Activinsights Ltd, United Kingdom) worn on the non-dominant wrist and recording with a sampling frequency of 50 Hz. Data on daily activity and sleep/wake cycles were analyzed with the GGIR package in R (v4.0.0) [13] based on validated intensity cutoffs with the following published values: light intensity physical activity (45.8-93.2 mg), moderate intensity physical activity (93.2-418.3 mg), and vigorous intensity physical activity (>418.3 mg) [14]. Other GGIR analysis parameters included an epoch length of 5 seconds, days defined from waking-up to waking-up, a valid day included ≥ 16 hours of data. From the GGIR sleep and physical activity analysis output, we extracted the sleep onset, wakeup time and sleep duration.

The CamNTech Actiheart device (version 4) assessed physical activity (activity counts (proprietary algorithm)), heart rate (HR, beats per minute) and heart rate variability (HRV using the root mean square of successive differences between normal heartbeats ($RMSSD^{-1}$) [15]). While the CamNTech Actiheart device is waterproof, participants were permitted to briefly remove the device during showers and baths. For technical reasons, data were recorded for 24h-72h at each time point.

Missing data were handled with a consistent set of criteria for each wearable data stream. For the MyFoodRepo data, we calculated the adherence as at least two ingestion events separated by at least 5 hours in a given day, as performed previously [16]. We report the eating metrics when at least 7 adherent days were recorded over a 2-week period. The CGM metrics were calculated when at least 7 consecutive days of data were recorded. In certain cases, the CGM device fell off during the recording period and was replaced, which occurred for two participants at baseline and two participants at follow-up. The MyFoodRepo data was analyzed alongside the CGM data in a previously published framework modeling data from multiple wearable devices (see Statistical Analyses and below) when both MyFoodRepo data and CGM data were concomitant and not missing [17]. GENEActiv data was used for subsequent GGIR analysis when at least 6 days of data were available, and we also filtered participants with more than 20% missing data. The Actiheart data was used when at least 48 hours of data was available i.e. for two full 24-hour cycles. Due to its positioning on the chest, the device could not be worn consistently by all breastfeeding participants. Upon data extraction, the Actiheart produces an estimated quality of the signal (range 0-1) and we filtered the data based on a threshold of 0.8.

Statistical analyses

Modeling ingestion events and glucose data. We combined the ingestion events with CGM data based on a recently published computational framework [17]. In short, the first assumption of the model is that calorie-containing meals can cause an increase in glucose levels before returning to steady state levels (due to glucose homeostasis, schematic in Fig. 1A). As the increase in glucose levels can vary according to the ingested food item, the relative glucose increase with respect to baseline levels is left as an inferred parameter for each unique meal, summarized as the "response height" (Fig. 1A) (meals with the same text annotation are given the same response height parameter

for simplicity). The length of time that the glucose takes to return to baseline levels is determined by a “response t½” parameter (Fig. 1A). Thus, each participant is conceptualized as a dynamical system that is trying to maintain glucose homeostasis in the face of external perturbations (e.g. food items and drinks). This set of parameters is learnt for each participant by the computational framework.

Even when postprandial glucose spikes caused by meals are accounted for in the model, the meal model alone can leave systematic 24-hour artifacts in the model fit (see ESM Results), and an underlying 24-hour cosinor function is therefore also included. The 24-hour cosinor function is described by the “baseline”, “amplitude” and “peak time” parameters (schematic in Fig. 2A), which are also learnt for each participant. The parameters of the model are estimated using Hamiltonian Markov Chain Monte Carlo (MCMC) using 4 different chains with 10,000 samples each (with a burn-in of 10,000 samples). We attempted to compare the peak time of the underlying glucose 24-hour rhythm between the two groups using a Watson-Williams test, but the peak times were too spread over the 24-hour clock for the test to be valid.

Longitudinal analysis. To compare the average longitudinal differences between baseline and follow-up across all participants, we pooled the GDM and non-GDM groups and used paired *t*-tests. These tests were performed using the ‘pingouin’ package (v0.5.3) within Python. To detect whether GDM status had an effect on the longitudinal difference between the baseline and the follow up visit, we use analysis of covariance (ANCOVA) with the following regression equation for each variable analyzed [18]:

follow up score = constant + a × baseline score + b × group (GDM=1, non-GDM=0),

which was performed using the ‘statsmodels’ package (v0.13.0) within Python), available from conda-forge at https://anaconda.org/conda-forge/statsmodels.

ESM Results

Lower amplitude of the underlying glucose 24-hour rhythm in the GDM group

When the glucose model was fitted to the CGM data using only ingestion events then the resulting model fit was inadequate (ESM Fig. 4A): the residual (i.e. the difference between the model fit and the data) showed a 24-hour cycle i.e. a systematic error in the model (ESM Fig. 4B). To resolve this, the glucose dynamics model needed an underlying glucose 24-hour rhythm during model fitting (ESM Fig. 4C), which produced a higher quality fit to the data (ESM Fig. 4D). This 24-hour rhythm has three parameters: a baseline parameter (i.e. the bottom of the trough), an amplitude describing the difference between the peak and trough, and a peak time (schematic in Fig. 2A).

Given that the 24-hour rhythm causes glucose fluctuations, one might naturally expect this 24-hour rhythm to drive increased glucose variability; however, we did not observe an association between this baseline trend and markers of glycaemic variability such as CV (ESM Fig. 4E) or MAGE (ESM Fig. 4F), presumably because the variance caused by this smooth trend is dominated by other sources.

Fasting glucose and cholesterol evolve during the postpartum period

Overall, the changes in metabolic parameters over the 6 months from baseline to follow-up were more marked than the differences between groups i.e., several variables showed longitudinal differences across all participants (ESM Fig. 5A), but there were no between-group differences in the changes (ESM Fig. 5B). The most marked difference was the decrease in total cholesterol (–0.67 mmol/L, 95%CI –0.96 to –0.38, $p < 0.001$, ESM Fig. 5C) and LDL cholesterol (–0.55 mmol/L, 95%CI –0.79 to –0.31, $p < 0.001$, ESM Fig. 5A). The waist circumference also decreased during this period (–4.3 cm, 95%CI –6.8 to –1.8, $p = 0.001$, ESM Fig. 5D).

In contrast, fasting plasma glucose increased over the postpartum period (+0.22 mmol/L, 95%CI 0.04–0.40, $p = 0.02$, ESM Fig. 5E), and an upwards trend in the CGM mean glucose ($p = 0.06$) and the baseline glucose level inferred from the glucose dynamics model accounting for underlying 24-hour rhythm and ingestion events ($p = 0.07$, ESM Fig. 5A). In terms of eating behavior, there was also a reduced percentage of fat consumption at follow-up (–2.1%, 95%CI –3.99 to –0.20, $p = 0.03$, ESM Fig. 5F).

ESM Figures

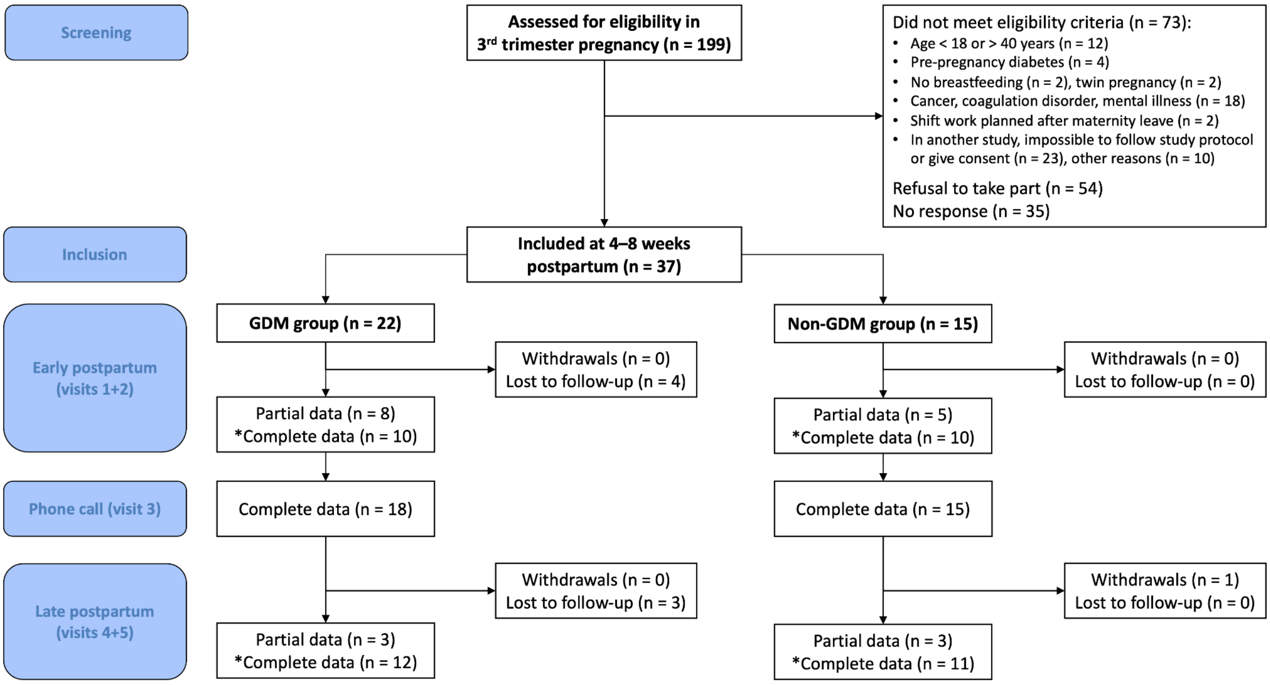

ESM Figure 1. Study design and recruitment of postpartum women into a prospective observational study, with reasons of exclusion and number of completed or partial data collection. * Data were considered complete according to these criteria: filled-in questionnaires on the electronic case report forms (RedCap), ≥ 7 days of food and drink consumption data with the smartphone app MyFoodRepo, ≥ 7 days of CGM data, ≥ 7 days of GENEActiv accelerometer data, ≥ 72 hours of Actiheart data (see text). *Abbreviations*: CGM, continuous glucose monitoring; GDM, gestational diabetes mellitus.

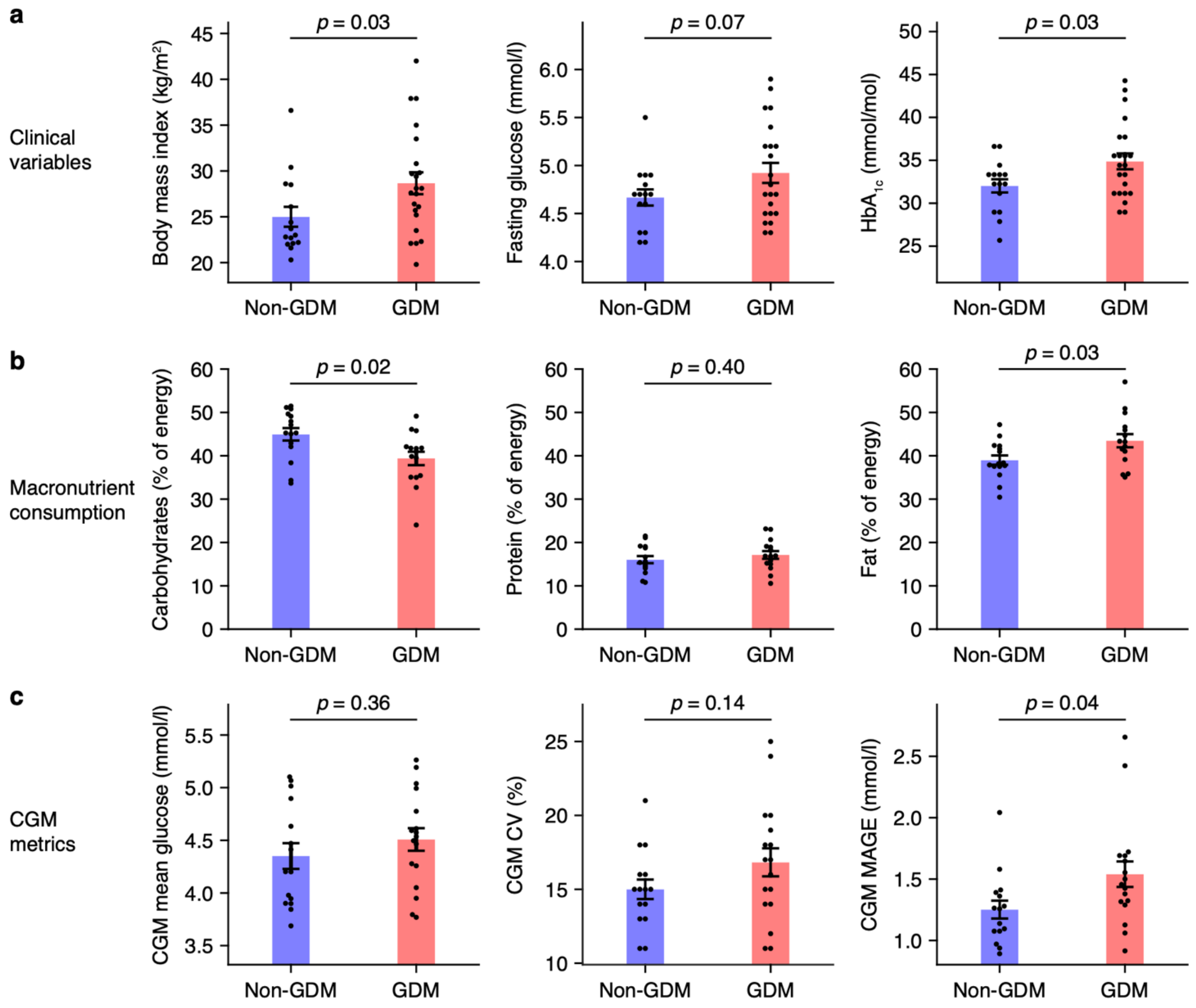

ESM Figure 2. Baseline comparison of key (a) clinical measurements, (b) macronutrient consumption expressed as % of the total energy intake recorded, and (c) CGM metrics between GDM (red) and non-GDM (blue) groups. P-values were calculated using the Welch separate variances t-test, except for MAGE which was compared with the Mann-Whitney U test. *Abbreviations*: CGM, continuous glucose monitoring; CV, coefficient of variation; GDM, gestational diabetes mellitus; HbA_{1c}, glycated haemoglobin; MAGE, mean amplitude of glycaemic excursion.

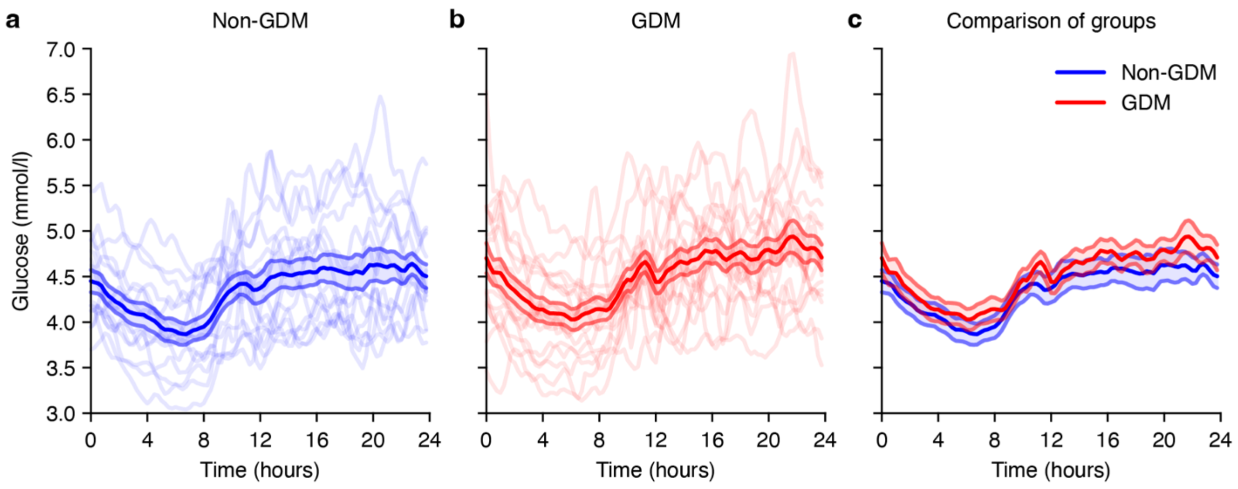

ESM Figure 3. 24-hour glucose profile comparison between non-GDM (blue) and GDM (red) groups. (a) In the non-GDM group, each light blue line represents the 24-hour mean glucose profile for one participant, taking the average value over all available days. The dark blue lines represent the group mean (and standard error) over all participants. (b) In the GDM group, each light red line represents the data from one participant, taking the average value over all available days. The dark red lines represent the group mean (and standard error) over all participants. (c) Direct comparison of the mean and standard error in both groups. *Abbreviation*: GDM, gestational diabetes mellitus.

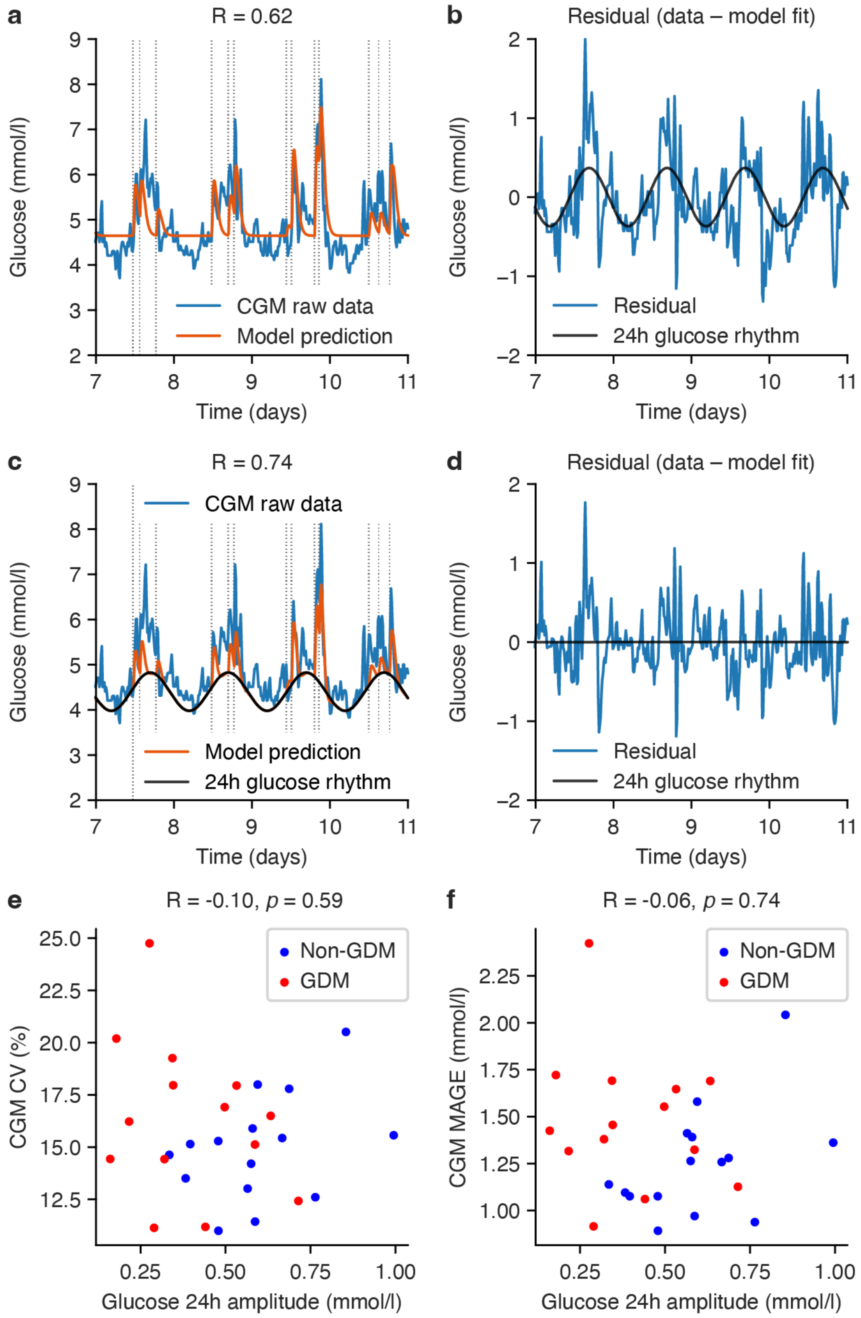

ESM Figure 4. Example of why adding a 24-hour underlying rhythm is necessary, which does not lead to overall higher glycaemic variability in the population (ID 05, non-GDM group). (a) We first try to fit a model of the CGM dynamics that only includes a contribution from each ingestion event, where only the recorded timestamps of food and drink ingestion are used to infer the size of the glucose spikes caused by meals. Blue, CGM raw data; dotted gray lines, the timestamps of ingestion events recorded with the smartphone app; orange, the model fit using the recorded timestamps. (b) The residual for the model (i.e. the data – model fit) that only uses ingestion events (blue), and a least squares fit of an oscillating 24-hour function (black). (c) A fit of the CGM data (blue) using a model (orange) that uses ingestion events as well as an underlying 24-hour function (blue). (d) The residual (i.e. the data – model fit) for the model that only uses meals as well as an underlying 24-hour function (blue), and a least squares fit of an oscillating 24-hour function (black). (e) The CGM CV as a function of glucose 24-hour amplitude in the non-GDM (blue) and GDM (red) groups. (f) The CGM MAGE as a function of glucose 24-hour amplitude in the non-GDM (blue) and GDM (red) groups. *Abbreviations*: CGM, continuous glucose monitoring; CV, coefficient of variation; GDM, gestational diabetes mellitus; MAGE, mean amplitude of glycaemic excursion; R, Pearson correlation coefficient.

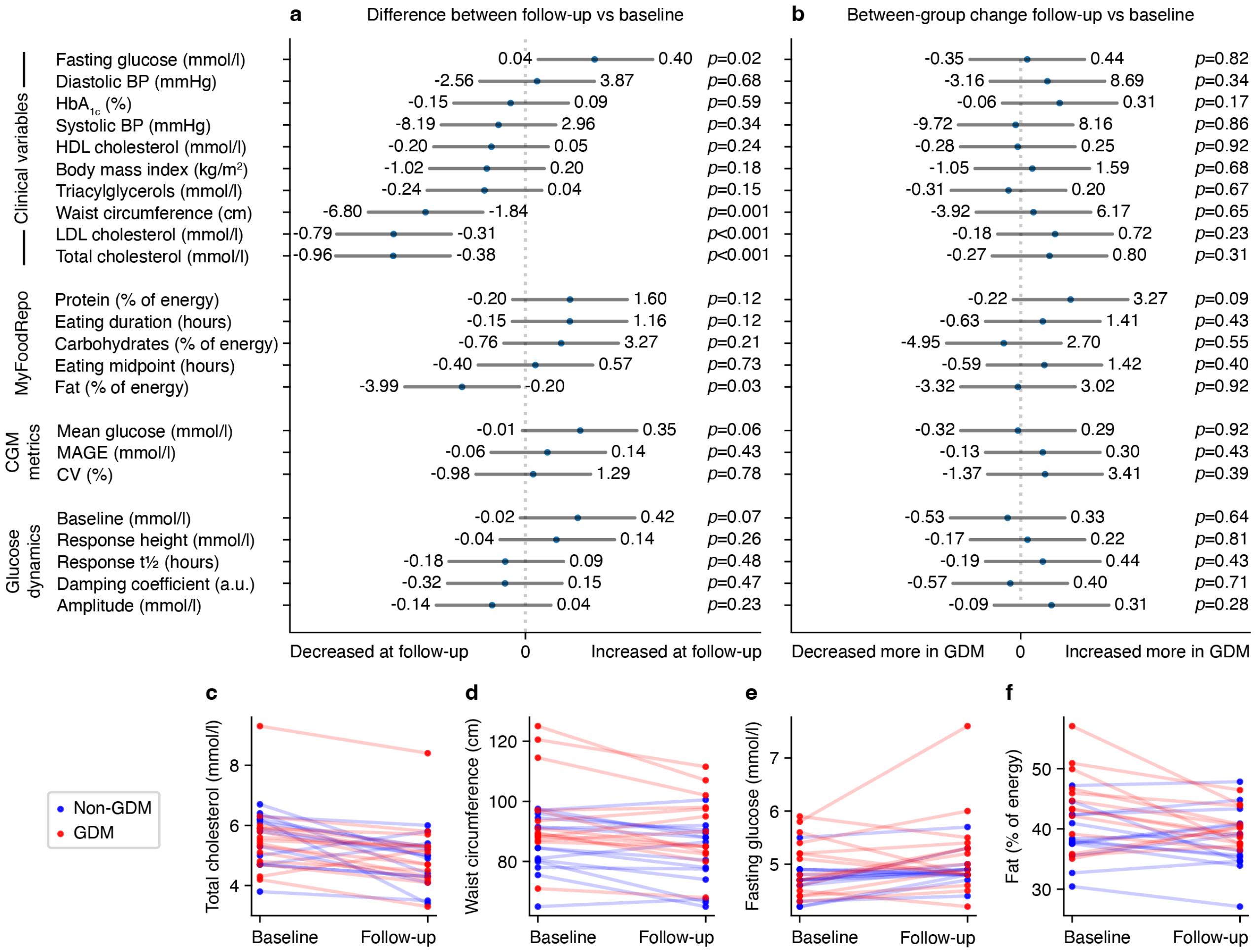

ESM Figure 5. Evolution of clinical measurements, eating patterns, CGM metrics and glucose dynamics model parameters from baseline to follow-up and between groups. (a) Means (dots) and 95%CI (bars) for the difference between follow-up and baseline, calculated using a paired t-test when data were available at the two study time points. Data points to the left of the vertical dashed line (difference = 0) denote a decrease at follow-up, and those to the right denote an increase at follow-up. (b) Means (dots) and 95%CI (bars) for the group effect of differences between follow-up and baseline, calculated using the analysis of covariance (ANCOVA). (c-f) Individual values at baseline and follow-up of selected variables, across the non-GDM (blue) and GDM (red) groups. Data were available for clinical variables (non-GDM: n = 14, GDM: n = 15); MyFoodRepo variables (non-GDM: n = 14, GDM: n = 13); CGM metrics (non-GDM: n = 13, GDM: n = 14); and glucose dynamics model parameters (non-GDM: n = 13, GDM: n = 11). *Abbreviations*: a.u., arbitrary unit, BP, blood pressure; CI, confidence intervals; GDM, gestational diabetes mellitus; HbA_{1c}, glycated haemoglobin; HDL, high-density lipoprotein; LDL, low-density lipoprotein; CGM, continuous glucose monitoring; CV, coefficient of variation; GDM, gestational diabetes mellitus; MAGE, mean amplitude of glycaemic excursions.

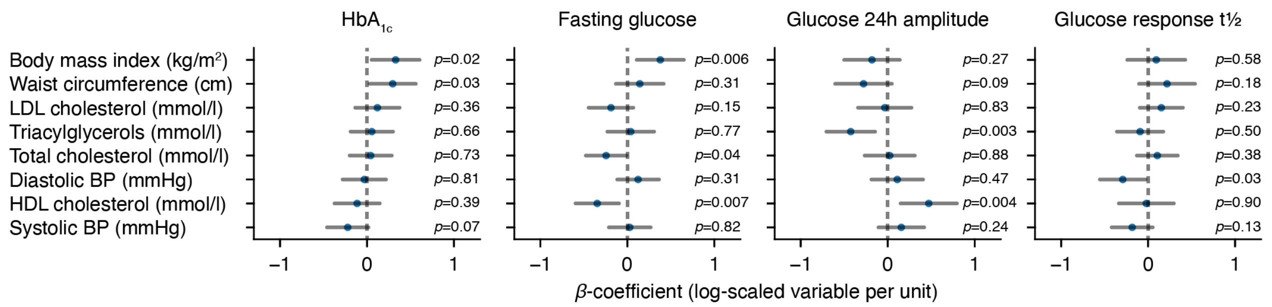

ESM Figure 6. In a linear mixed model pooling all baseline and follow-up data, glycaemic variables are associated with other metabolic markers (i.e. from the definition of the metabolic syndrome). Points show the inferred regression coefficient, horizontal lines the 95%CI, and p-values at the right of each plot. The dependent variables were log-transformed (natural log). *Abbreviations*: BP, blood pressure; CI, confidence interval; HbA_{1c}, glycated hemoglobin; HDL, high-density lipoprotein; LDL, low-density lipoprotein.

ESM References

1. Craig CL, Marshall AL, Sjöström M, et al (2003) International physical activity questionnaire: 12-country reliability and validity. Med Sci Sports Exerc 35(8):1381–1395. https://doi.org/10.1249/01.MSS.0000078924.61453.FB
2. Buysse DJ, Reynolds III CF, Monk TH, Berman SR, Kupfer DJ (1989) The Pittsburgh Sleep Quality Index - a New Instrument for Psychiatric Practice and Research. Psychiatry Res 28(2):193–213. https://doi.org/10.1016/0165-1781(89)90047-4
3. Horne JA, Östberg O (1976) A self-assessment questionnaire to determine morningness-eveningness in human circadian rhythms. Int J Chronobiol 4(2):97–110
4. World Health Organization (2011) Waist circumference and waist-hip ratio: report of a WHO expert consultation, Geneva, 8-11 December 2008. World Health Organization, Geneva
5. American Diabetes Association Professional Practice Committee (2024) 2. Diagnosis and Classification of Diabetes: Standards of Care in Diabetes-2024. Diab Care 47(Suppl 1):S20–S42. https://doi.org/10.2337/dc24-S002
6. Zuppinger C, Taffé P, Burger G, et al (2022) Performance of the Digital Dietary Assessment Tool MyFoodRepo. Nutrients 14(3):635. https://doi.org/10.3390/nu14030635
7. Héritier H, Allémann C, Balakiriev O, et al (2023) Food & You: A digital cohort on personalized nutrition. PLOS Digit Health 2(11):e0000389. https://doi.org/10.1371/journal.pdig.0000389
8. Gill S, Panda S (2015) A Smartphone App Reveals Erratic Diurnal Eating Patterns in Humans that Can Be Modulated for Health Benefits. Cell Metab 22(5):789–798. https://doi.org/10.1016/j.cmet.2015.09.005
9. Zahedani AD, Veluvali A, McLaughlin T, et al (2023) Digital health application integrating wearable data and behavioral patterns improves metabolic health. npj Digit Med 6(1):1–15. https://doi.org/10.1038/s41746-023-00956-y
10. Phillips NE, Mareschal J, Schwab N, et al (2021) The Effects of Time-Restricted Eating versus Standard Dietary Advice on Weight, Metabolic Health and the Consumption of Processed Food: A Pragmatic Randomised Controlled Trial in Community-Based Adults. Nutrients 13(3):1042. https://doi.org/10.3390/nu13031042
11. Olawsky E, Zhang Y, Eberly LE, Helgeson ES, Chow LS (2022) A New Analysis Tool for Continuous Glucose Monitor Data. J Diabetes Sci Technol 16(6):1496–1504. https://doi.org/10.1177/19322968211028909
12. Service FJ, Molnar GD, Rosevear JW, Ackerman E, Gatewood LC, Taylor WF (1970) Mean amplitude of glycemic excursions, a measure of diabetic instability. Diabetes 19(9):644–655. https://doi.org/10.2337/diab.19.9.644
13. Migueles JH, Rowlands AV, Huber F, Sabia S, van Hees VT (2019) GGIR: A Research Community–Driven Open Source R Package for Generating Physical Activity and Sleep Outcomes From Multi-Day Raw Accelerometer Data. JMPB 2(3):188–196. https://doi.org/10.1123/jmpb.2018-0063
14. Hildebrand M, VAN Hees VT, Hansen BH, Ekelund U (2014) Age group comparability of raw accelerometer output from wrist- and hip-worn monitors. Med Sci Sports Exerc 46(9):1816–1824. https://doi.org/10.1249/MSS.0000000000000289
15. Shaffer F, Ginsberg JP (2017) An Overview of Heart Rate Variability Metrics and Norms. Front Public Health 5:258. https://doi.org/10.3389/fpubh.2017.00258
16. Chow LS, Manoogian ENC, Alvear A, et al (2020) Time-Restricted Eating Effects on Body Composition and Metabolic Measures in Humans who are Overweight: A Feasibility Study. Obesity 28(5):860–869. https://doi.org/10.1002/oby.22756
17. Phillips NE, Collet T-H, Naef F (2023) Uncovering personalized glucose responses and circadian rhythms from multiple wearable biosensors with Bayesian dynamical modeling. Cell Rep Methods 3(8):100545. https://doi.org/10.1016/j.crmeth.2023.100545
18. Vickers AJ, Altman DG (2001) Statistics notes: Analysing controlled trials with baseline and follow up measurements. BMJ 323(7321):1123–1124. https://doi.org/10.1136/bmj.323.7321.1123
